# How can students contribute? A qualitative study of active student involvement in development of technological learning material for clinical skills training

**DOI:** 10.1186/s12912-016-0125-y

**Published:** 2016-01-12

**Authors:** Cecilie Haraldseid, Febe Friberg, Karina Aase

**Affiliations:** Department of Health Studies, University of Stavanger, 4036 Stavanger, Norway; Sahlgrenska Academy, Göteborg University, Gøteborg, Sweden

**Keywords:** Clinical skills, Nursing education, Technology, User involvement, Student involvement

## Abstract

**Background:**

Policy initiatives and an increasing amount of the literature within higher education both call for students to become more involved in creating their own learning. However, there is a lack of studies in undergraduate nursing education that actively involve students in developing such learning material with descriptions of the students’ roles in these interactive processes.

**Method:**

Explorative qualitative study, using data from focus group interviews, field notes and student notes. The data has been subjected to qualitative content analysis.

**Results:**

Active student involvement through an iterative process identified five different learning needs that are especially important to the students: clarification of learning expectations, help to recognize the bigger picture, stimulation of interaction, creation of structure, and receiving context- specific content.

**Conclusion:**

The iterative process involvement of students during the development of new technological learning material will enhance the identification of important learning needs for students. The use of student and teacher knowledge through an adapted co-design process is the most optimal level of that involvement.

## Background

Clinical skills training is a fundamental part of nursing education wherein students combine sensory, motor and cognitive learning processes and learn how to perceive and act in any situation presented to them [1]. This complexity of clinical skills acquisition demands a range of different learning approaches for nursing students to learn what they need to know [2]. A shift toward more learner active teaching strategies in higher education [3] and an expanding knowledge of information technology [4] has produced many changes in clinical skills training over the last few years. This change has produced multiple new learning strategies, such as simulation, serious games, online learning material, and personal digital assistants, which have emerged and become part of nursing student clinical skills training [5–9]. Nevertheless, the quest to determine the most optimal learning method within clinical skills is still being sought by many nurse educators [10].

Further, new policy initiatives and an increasing amount of the literature within higher education call for students, not only to be consulted during the development of learning strategies, but also become actually involved as co-designers, co-producers, and co-creators of their own learning [11, 12]. The goal is to place student needs at the center of the design process [13] and thus view the student as a knowledgeable and critical partner in learning [14]. While the idea of user involvment already is an established best practice within health care services [15–17] nursing education has only to some extent actually embraced this student collaboration concept [18, 19]. Student experiences have, however, been deemed valuable for future educational improvement [20] and student involvement has been used in some curriculum design [21, 22]. There is also some comprehensive literature on student use, benefits, barriers, and their experiences with already developed programs and devices [23–25]. On the other hand, there is a shortage of literature on active involvement of nursing students in the actual development processes and especially a lack of descriptive studies that examine the actual personal role of the students when they are engaged in the creation of their own learning activities [26]. In Norway, undergraduate nursing education follows the Bologna requirements with 3 years of full-time study resulting in a bachlor degree [27]. Student involvement is ensured through law [28] where the minimum requirement is yearly student evaluation of the educational programme provided by the institution. The Ministry of Education also requires the educational institutions to gear their educational approach to the ‘*active, participating student’*, through a White Paper submited to the Norwegian Parliament [29]. While these official documents have ensured some participation, the room for individual interpretation of its execution often results in the use of representatives rather than participatory or prefigurative forms [30].

### Aim of the study

The aim of this study was to explore and describe the actual process of student involvement when developing technological learning material for clinical skills training in a Norwegian nursing faculty. Two research questions were developed for this purpose:

- How can nursing faculties actively involve their nursing students in the process of developing technological learning material?
- How can both students’ roles and contributions in the development process of such technological learning material be best described?

## Methods

### Design

The study was grounded in the idea of user involvement and the methodology of participatory design (PD). PD builds on the line of reasoning that key to finding the gaps that matters lies in involving the end users in development and design of services [31]. The process entails actively involving a group of people and bringing them to a consensus on what they want to do and how best to do it. In order to meet the actual needs of the users, their involvement must be incorporated into both design and development [31]. Through this process, PD has the potential of increasing the ease of implementation and of creating the benefits of credibility and legitimacy, while ensuring that the final design truly meets the precise needs of its users [19]. The approach has been specially suggested for use within educational settings due to its ability to take student perspectives into account [11]. While similar approaches such as experience-based co-design (EBCD) offers a series of stages to follow [32, 33] PD does not entail an specific description of how to involve the end users in the development process, but rather focuses on the involvement itself. In this study, the methods of data collection therefore needed to both actively and creatively engage the students in the developmental process, while giving the researchers the opportunity to grasp the students’ perspectives throughout the developmental process. An explorative qualitative approach was chosen as appropriate for arriving at an in-depth understanding of human behavior, by giving the participants room and opportunity to describe and explain their own experiences [34]. The development process was elaborated by the authors of the paper and divided into five phases; (1) initial phase, (2) Investigation phase, (3) revision phase, (4) exploratory test phase, and (5) finalization phase. The students contributed to different activities and to the collection of different data throughout the development process. An overview of activities and data collection is found in Table 1.

**Table 1:** Overview of research activities and collection of data material for study

Phase	When	Where	Activity	Data material	Participants
Initial Phase	Autumn 2013	CSL on campus	Tablets borrowed 134 times for unsupervised training sessions	Field notes	165 students
Investigative Phase	Spring 2014	Meeting room on campus	Two focus group Interviews	Transcription of interviews and field notes	Group A: 11 students
					Group B: 8 students
Revision Phase	Spring 2014	First author’s office at campus	Four revision meetings E-mail exchanges	Field notes from the meetings, taken by first author	First author, clinical nurse specialist, faculty teacher, and Senior interaction designer
Exploratory test Phase	Spring 2014	CSL on campus	Two practical test sessions	Student notes during practical test	Group C: 5 Students
					Group D: 6 Students
		Meeting room on campus	Two focus group Interviews	Transcription of interviews and field notes	Group C: 5 Students
					Group D: 6 students
Finalization Phase	Spring 2014	First author’s office	One revision meeting	Field notes	First author, faculty teachers

### Contextual setting

The technological learning material was applied to the clinical skills course at a Norwegian nursing faculty to teach undergraduate nursing students the 13 clinical skills required to pass that course. The course the technological learning material was applied to was based on a combination of supervised and unsupervised practice sessions. There were nine different supervised training sessions wherein a teacher-led group of 10–12 students practiced the 13 different scenarios. In addition, the students were given unlimited access to the Clinical Skills Laboratory (CSL) at the campus where they were expected to administer their own unsupervised training sessions. At the end of the course, all students were tested in one of the 13, randomly chosen skills in practical oral examination. For details of the course and the CSL environment, see C Haraldseid, F Friberg and K Aase [35]. Portable SimPad® tablets were used as technological mediators of the offered learning material. The main features of the tablet was; preprogramming correct actions that could be taken, feedback on actions taken, and to linking actions to responses. The user was thereby guided through a scenario, which could develop in multiple ways, as different actions might result in different outcomes. The software also gave the user a log of their actions at the end of each scenario and the programmer had the opportunity to add log comments, give the instructor instant messages, or set time limits for when actions needed to take place. By pre-programming the tablets, the students were able to run the required scenarios on their own.

Prior to involving the student in the developmental process actively, four prototype scenarios were developed by a teacher team to exemplify for the students how the features of the tablet could be used. To demonstrate to the students what they were asked to do, all students (165) enrolled in the clinical skills course were given a 1-hour introductory instruction lecture on how to operate their tablets, including the possibility of testing the device in groups. The prototype scenarios were also made available for use during two compulsory, supervised training sessions where the students had the opportunity to access their tablets during unsupervised training sessions to test the scenarios and become comfortable with their use. After the introduction, the students were involved in different phases and in different activities as shown in Table 1.

### Study participants

The study was undertaken at a Norwegian nursing faculty during Fall 2013 and Spring 2014 terms. In the Initial phase, all students enrolled in the clinical skills course were informed of the ongoing project and had the opportunity to familiarize themselves with the tablets and use of them as desired and when and how they wanted. The students participating in the Investigation and Exploratory Test phase were recruited during the initial phase through purposive sampling among all 165 students. This recruitment was done after the students completed their clinical skills course. Due to their participation in the course these students would have important experiences of their needs and the challenges that would present during clinical skills acquisition, together with first -hand user information on how the prototype of the learning material used in the course could be improved. The students were recruited by the first author through an open invitation in one of the faculty lecture classes. All students wishing to participate were encouraged to approach the first author personally or via an e-mail after class. There were no prerequisite for how much the students used the prototype of the learning material during the course, as those without excessive experience with the tablets could also contribute with important experiences leading to improvements. In total, 19 students contributed to four focus group interviews and two practical training sessions. In their own reporting, five of these 19 students stated they had used the tablet ‘*a little’,* six had used it ‘*some*’, and eight reported they had used it ‘*a lot*’. During the focus groups in the Investigation Phase the 19 students were divided into two groups with eight students in Group A and 11 in Group B. The division into the groups were based on the students’ schedules and their convenience. In the Exploratory Test Phase, 11 out of the original 19 students who participated did so based on availability with five from Group A and six from Group B. These 11 students were then divided into Groups C and D (see Table 1).

During the Revision and the Finalization phases, the first author organized meetings and conducted the process of making changes to the learning material. A clinical nurse specialist from the hospital contributed as a direct result of the students’ feedback, and two faculty teachers were consulted to make sure the current alterations matched best practice guidelines and required course content. A senior interaction designer was consulted on how to integrate the students’ feedback to the technological choices available on the tablet set-up.

### Ethical considerations

During the Initial phase of the study the students were given oral information about the ongoing project, confirming that participation was voluntary, which is in line with the principles outlined in the Declaration of Helsinki (World Medical Association Declaration of Helsinki, 2005). The students who proceeded to participate in the Investigation Phase and the Exploratory Test Phase received both written and oral information on the background and goal of the study, including information about their right to withdraw from the study at any point during it. Written informed consent was collected prior to data collection in both the Investigation Phase and the Exploratory Test Phase. Since the current research study involved no medical interventions or collection of health related information, the approval authority is the Norwegian Social Science Data Services (NSD) who also assess the ethical aspects of recruitment and informed consent. Approval for the study was therefore obtained from the NSD (Reference Number. 36260) and from the head of the nursing faculty.

### Data collection

The data in the study was collected through field notes and focus group interviews. Field notes were collected through informal meetings between the first author and the students, for example, during the delivery and return of the tablets or if any students approached the first author with comments about tablet use. In addition, notes were taken at all revision meetings, and the students took their own notes during the practical test session.

All focus group interviews were conducted in a meeting room on campus. Interviews with Groups A and B were conducted 6 weeks after these students completed the clinical skills course, while the interviews with Groups C and D were conducted 9 weeks after their completion of the course. All interviews were moderated by the first author and assisted by the third author. Interaction between the students was encouraged with the moderator asking, prompting, and clarifying questions. Interviews were audio recorded while both the moderator and assistant moderator wrote field notes to complement the audiotaping. The Investigation Phase and the Finalization Phase had their own separate aims and interview guidelines, respectively.

In the Investigation Phase, the focus group interviews [36] lasted for 60–80 min. The goal was to explore the students’ requirements during unsupervised training and how the technological learning material could contribute to fulfilling their learning needs. The interviews commenced with general questions about the students experiences in CSL training. Once the students seemed comfortable with the interviewers, the questions gradually turned to the theme of the study [36]. Those questions pertained to the issues the students enjoyed or found difficult in the CSL environment, their needs, and how their training could be improved.

The Exploratory Test Phase consisted of both practical training sessions and focus group interviews. The training session lasted for 45–60 min. In that session the students received a revised version of the technological learning material, based on the needs and feedback gathered during the Investigation Phase. They were given all the necessary equipment to complete the training session. The students were divided in groups of two or three and instructed to test the device as it suited them, but they had to complete the entire scenario. They were encouraged to take breaks in the scenario and discuss the process with each other, while taking notes of what they had experienced, felt and thought. Immediately after the practical training session, the groups were gathered for joint discussion in focus group interviews. The focus group lasted for approximately 30 min. It attended to different aspects of the learning material, in particular, the layout, the content, and areas that needed improvement and ways to undertake such improvement. In addition, the students handed in their personal notes from the practical test session for use as supplementary data material.

### Data analysis

The main topic for analysis was the focus group interviews, while the field notes and student notes were used as supplementary data material. Qualitative content analysis was chosen as the method to analyze and categorize data [37]. All interviews were transcribed by the first author 1 or 2 days after the interviews. The transcripts and field notes were also analyzed and coded by the first author. In the first step, the data was read as openly as possible, trying to get an impression of both parts and the whole. In the second step, after reducing the number of words while still preserving the content, the meaning units were shortened and coded. This step compared the units and sorted the text into relevant themes [37]. As the authors reviewed and discussed these themes, it became clear that several themes were overlapping, so some themes were merged at a more abstract level in Step 3. Step 4 consisted of reading the field notes and interview transcriptions again, making sure the final themes covered the whole picture. During this step it became clear that the themes represented five different learning needs that were especially important for the students: clarification of learning expectations, help to recognize the bigger picture, stimulation of interaction, creation of structure, and receiving context-specific content. To establish trustworthiness throughout the entire study, the first and third authors conducted the interviews and took all the field notes, while the second author formulated the critical questions needed to expand the understanding of the gathered data [37, 38]. Different interpretations found during the analytical steps were repeatedly discussed and reinterpreted by all authors together.

## Results

Through a process of actively involving nursing students in the development of technological learning material, their role evolved into being advocates for learning needs that are necessary for tailoring their learning material accordingly. While the nursing faculty staff may hold the key to what students should learn, the students described how their learning could be most constructively achieved. These learning needs were not initially explicitly described, but rather evolved over time as a result of the iterative involvement. By systematically collecting the students’ experiences and using different data sources, their learning needs became both explicit and concise. These learning needs were subsequently used as the basis for identifying the practical implications and changes to be made to the technological learning material. The five themes evolved through the process of the material development and represent the students’ different learning needs.

### Clarification of learning expectations

The students undertook a range of different actions to prepare themselves for the final exam, among these were multiple choice questions, video films, assigned reading and correspondence with teachers through e-mails, online discussions forums, and personal meetings. While these different actions did serve different needs, the students’ main goal was to understand what the faculty teachers actually expected of them in terms of learning. Their time and energy were often used to decipher the *real* or hidden meaning behind the information and questions they received from faculty teachers. This often led to uncertainty: ‘*If you don’t have the answer, then we go back and forth. What do they* mean*? What do* they *think? How do* you *interpret it? Then you are left with three different answers…then this uncertainty appears (Interviews, Group B)*. These were all typical questions from the students. Their biggest fear was a failure to grasp what they needed to learn, which would result in their failing the exam. This fear left them uncertain and insecure, indeed more worried about what the *faculty* wanted them to know than about how they could learn better and understand the different aspects of the actual procedure. ‘*The students ask a lot of questions over and over again, and need detailed conformation and information about* what *to learn (Field notes)*. The students, therefore, needed better preparation and more information about their teachers’ expectations. By clarifying expectations, important time and energy could be diverted toward achieving specific learning goals, instead of searching for them. When addressing this issue by integrating learning goals into the learning material, the students found it easier to grasp what was expected of them, as ‘*it stood there, in black and white: what is expected of you and what is the answer*’ *(Interview, Group C).*

### Help to recognize the bigger picture

Another issue that claimed much of the students’ attention was the variety of answers they could find for what they saw as being the same type of questions. In their struggle to find the ‘right’ answers, they often consulted different sources of information, resulting in them finding more discrepancies than clarification. For example, ‘*…we ask the same question to different teachers and get different answers’ (Interview, Group B)*. It seemed that the novelty of their profession led to an extreme attention to details, focusing more on the pieces of the puzzle than the big picture. They seemed to be self-aware of their own deficiency in recognizing the bigger picture while lacking the tools to do something about it ‘…*there is probably many ways to Rome, and they are all right, but we cannot see all the possibilities. For us there is so much we need to keep in mind; it is this procedure and this procedure, we cannot see all the possibilities, we need it to be more specific; that’s how it is. Maybe it sounds kind of square, but that’s how it is!*’ *(Interview, Group B).* While all these small variations were a source of frustration, their biggest issue was the differences between actual practice and what was taught at the faculty: ‘*I have practiced (on the procedures) the way I think the sensors would like me to solve the task at the exam, in order to pass. You need to know how it’s supposed to be done when you come in there (to the school exam) because* t*he reality in the CSL is not exactly the same as the reality we meet when we are on prac*’ *(Interview, Group A).*

The students therefore wanted answers that ‘belonged’ to every question and a recipe for how things were done and why. While the students searched for ways to simplify their quest for what they saw as ‘right answers’ the field notes also speculated that the real issue was understanding the bigger picture and indeed, ‘*recipes with belonging arguments of ‘why’ could help students think picture instead of pieces? (Field Notes)*. The original questions embedded in the learning material were therefore complemented with answers and arguments. This aimed to help the students better understand the whole scenario, seeing better connections between principles, actions and arguments: *‘(…) I think more now, I pay attention if the doctor (when in prac) does it correctly (…) Before I never had the knowledge to do that’ (Interview, Group B).*

### Stimulation of interaction

Besides helping to recognize the whole picture and clarifying expectations, the students appeared to seek, and value every possibility for more interaction. Types of interactions varied between students and those between students and teachers. What all of the activities had in common, however, was that they gave the students’ the ability to challenge their own knowledge, test their knowledge, and rate their knowledge to the knowledge of others and thus progress. While all forms of feedback were sought, teacher feedback was especially valued. The students saw this feedback as the safest source of information and information of the highest level to test their knowledge against, although it was often the least available option. The most used alternative was to practice, discuss, and receive precise feedback through group interaction with other students. The problem with this process, however, was uncertainty about the quality of the feedback coming from their peers: ‘*it is okay to ask each other, I might ask Mary, and then she answers and I think* “hm…yes I’m satisfied with that answer”, *but sometimes I think* “is Mary right?…is that the right answer?” *And then you get hesitant, because we are not professionals any of us! So sometimes it would be great to have a teacher here!’ (Interview, Group D).*

The tablet, however, could be used to ask stimulating questions and give feedback that would trigger more interaction both between the students and the tablet and between the students who were practicing together. Critical questions created enthusiasm and engagement with the procedure, while also eliminating the uncertainty that could be raised between peers as in *‘you know that what you learn is correct’, ‘it’s a quality assurance’ (Interview, Group B).*

While the prototype scenarios entailed a limited number of questions, one of the later versions integrated questions into almost every answer to test how the student responded. As noted in the Field Notes, there was*’A surprising enthusiasm about all the questions in scenario 4 (Field Notes)’.* This mood seemed to be explained by the fact that the students saw the questions as a chance to be challenged about aspects of the procedure that they had not thought of, to ‘get *some aha-experiences for ourselves (Interview, Group A)’* and also to receive tips for possible questions for the exam. All these characteristics, taken together, made the tablet interesting as a potential element for creating highly valued interactions among the students that helped them both prepare and learn.

### Creation of structure

Training for the practical oral exam was seen by the students as a stressful event. While they valued all sorts of tools that could help them during training, it was important that these tools simplified, instead of complicating, their preparations. Simplicity, overview, and structure were thus keywords found in the students’ feedback created through the layout and design of the content on the tablet. It was important that ‘*for someone that is doing this for the first time it should not feel so overwhelming’, (Interview, Group C).* Student feedback, therefore, led to scenarios that were structured chronologically, dividing the different tasks into separate sections to create a natural progression in the scenario. While this dissection could be seen as fragmenting the bigger picture, it accommodated the students’ previous statements about needing a recipe to follow due to the novelty of their profession: ‘*in nursing there is so much (to know)…. But now it gets taken down a notch, and it gets easier to act accordingly*’ *(Interview, Group B).* Using the same basic structure in all scenarios created a sense of familiarity and predictability for the students, while giving them the structure they needed. Another important aspect for creating such a structure was enabling the students to follow it. The initial lack of attention to details often caused a gap between what teachers believed was communicated and what the students *perceived* as having been communicated. *‘People are more amateurs than you think (…) I remember when we first started here (in the CSL) some of us had never measured a blood pressure before, and then you are presented with a film, and you see how they measure, but there is no sound. Yes, you blow up* this *and you put* these *in your ears, but you don’t know how it is supposed to sound. It’s like if I was to teach you how to bake a cake I could say:* “then you take the flour…” *but you would want to know how much flour to take wouldn’t you?! (Interview, Group B).*

This attention to detail often made the scenarios information rich and long, something that also claimed an opportunity to navigate back and forth in the scenarios and check information they were unsure of, while also to making it easier for them to repeat specific sections of the scenario while creating the structure. The students also pointed out where information needed to be elaborated on, what information could be misjudged or misunderstood, and how information should be phrased, thus keeping them truly on track to know what was important and avoid potential confusion.

### Receive context-specific content

While creating a structure revolved around how information was given, the students’ contributions were also concerned with what kind of information they needed. Multiple learning tools competed for their attention, and the trouble of their not knowing the best way to learn caused them to jump from one remedy to another. What made them favor the learning material on the tablet, however, was that the content could be specified to each context and situation. Disputes and frustration seemed to be more related to questions concerning context. Discrepancies in answers and information often were rooted in the fact that they were given for different contexts. By giving and explaining context specific information, more tailored to the scenarios, the process helped settle disputes rather than create more of them.

That the learning material was produced in collaboration with teachers and a clinical nurse specialist created a new coherence between what happened during practice, was written in the referenced literature, lectured about in class and the information stored on the tablet. Taken together, this process clarified several factors that had previously been seen as discrepancies by the students, and it helped them see that instruction could be done differently, depending on the context: (Student 1) ‘*then we actually get an answer….’ (Student2) Instead of just us students discussing, because then we never get answers…(Interview, Group B)’*. Training using the tablet also created an unexpected positive aspect that helped them prepare for the more psychological aspect of the exam: *This is a very good way to work. You get kind of nervous, get some performance anxiety, because you know that she has something that resembles the exam (the tablet). You get to practice the exam situation in a systematic way (Interview, Group C)’.* Making the instruction context specific also meant challenging students to think about the context. Asking for explanations and reasons for their actions in each specific setting, but also asking what would have changed if something in the context was changed: *‘By using the Simpad, I got quite a few extra tips about the questions that might come, what the sensors could ask, it made me become more aware of the reasons behind things (Interview, Group A) …*’.

### Practical implications

**Fig. 1:**
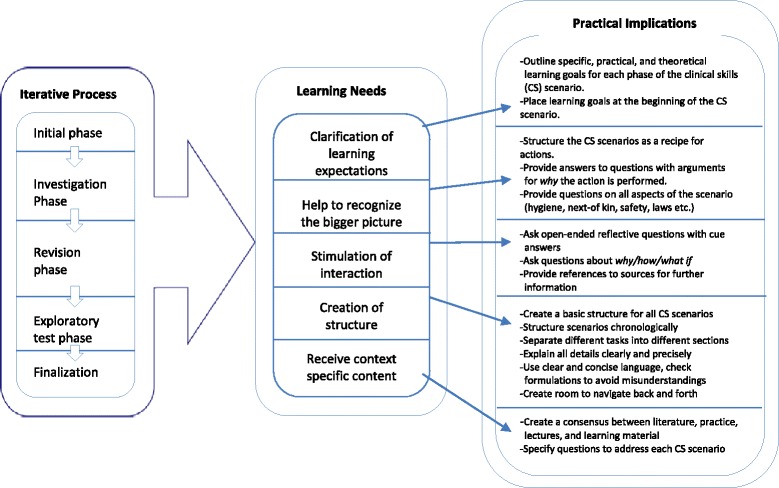
From student involvement to practical implications

In order to operationalize the findings for future development of technological learning material, the five different learning needs that evolved through the iterative student involvement process were linked to a set of practical implications. These practical implications can be seen as a checklist of important aspects to consider for future development of technological learning material. The implications are structured in a figure indicating the relationship between the iterative student involvement, the evolved student learning needs, and the practical implications (Fig. 1).

As Fig. 1 displays, each of the five identified learning needs can be operationalized through a set of different implications. It is important to remember that the iterative student involvement process entailed student validation of all implications in this study, and the findings may vary by context. In addition, several aspects related to students’ involvement need to be considered, some of which are discussed in the following.

## Discussion

This paper documents how nursing students can be actively involved in the development of their own learning materials and how their role indeed contributed to the identification of five different and important learning needs. In the following discussion we look at the involvement when using an iterative process and the level of student involvement for the best learning outcomes.

### Using an iterative process for involvement

The students in this study were actively involved in several phases throughout the development process. The process was iterative and entailed identifying student needs and trying to meet them, before adjusting both the needs and the solutions. Without this repetitive process, the unveiling of the specific learning needs would have been more difficult. One of the most important catalysts that enables human beings to become proactive and engaged in activities according to RM Ryan and EL Deci [39] are the catalyzing factors in their environment. Among these are autonomy, which plays a vital role in human motivation [40]. Facilitating autonomy demands decreases external control, provision of individual choices, and acknowledgement of feelings [41]. We believe that the iterative process in contrast to a single mapping of students’ experiences facilitates autonomy through acknowledging and integrating students’ thoughts and feeling over time. Parallels can be drawn to Freire’s [42] deliberative pedagogy where creativity and participation are taken into account. This choice again made the students in this study engaged and interested in the possibility of being able to influence their own learning material.

The process of iterative student involvement can be difficult to achieve due to limited time and resources. Teachers also often experience anxiety over reduced authority when they open up to students for feedback on their performance [43]. Further, students may feel insufficiently equipped to participate in the process [44]. On the other hand, engagement and student involvement, once undertaken, makes students more aware of their faculty’s commitment to their own learning [45], thus enhancing knowledge of their own learning process [46], playing an important role in quality improvement [44] and increasing student satisfaction with the material provided them. While satisfaction should not be equalized with quality [47], dissatisfaction with teaching has negative effects on both motivation and engagement [48]. The results from this study indicate that iterative processes that do identify students’ needs assumable can foster more motivation and engagement and have the possibility of ensuring the development of learning design that satisfies students’ needs.

### Level of student involvement

Although user involvement is deemed to be beneficial, there is ongoing debate concerning the extent of that involvement. C Bovill and CJ Bulley [12] adapted Arnstein’s ladder of citizen participation [49] to revolve more around student involvement, and specifically distinguish between ‘tutors in control’ and ‘students in control’. The highest level of participation is when students themselves control decision-making and have substantial influence, while the lowest level of participation is when there is no student participation [12]. The highest level of involvement removes the teacher from the equation, leaving the students absent from the influence of the tutor. While this active participation can bring to bear a high level of autonomy, as supported by EL Deci and RM Ryan [41], the removal of the tutor is still challenging in the higher education context due to quality assurance systems [12]. It could also be directly unwise sometimes, as the qualities of good teachers are still vital for the facilitation of learning according to J Hattie [50]. Striving for student participation at the highest rung also was contradicted by some of our findings. Our students clearly stated that the role of the teacher was important, as they needed clarification of learning expectations, along with questions, clues, and answers to help them see details they were not able to see for themselves. The teacher is, therefore, important when designing technological learning material and is supported by PA Kirschner [26]. Shared involvement in the overall process makes both students and teachers valuable, where the aim is not necessarily simply to strive to reach the highest rung of the ladder. Within other professions, user involvement and participatory approaches have gradually shifted toward similar approaches such as ‘co-creation’, ‘co-design’ or ‘experience-based co-design’ [51–54]. These methods reflect a more democratized approach where the different stakeholders are united in a partnership agreement that fosters a bottom-up approach [33]. The idea is to involve all parties in an ongoing creative process, giving end-users a larger role and the power to make decisions [51]. Education, as advocated by Paulo Freire should in itself be an empowering, participatory process [42]. Involving students through co-creation and co-design could therefore seem suitable for the educational setting since participation and empowerment are the direct consequences of this process. Although the literature on co-creation and co-design within education is somewhat scarce, the method has proven fruitful in areas like health care and service improvement [55–57]. Collaboration through combining experience, creativity, and engagement of both students and teachers in a co-design of technological learning material could therefore be beneficial for in many respects.

Although different learning styles are believed to suit different students, the focus of this study was not to match a specific style to a particular type of students but rather to add to the body of learning materials in order to increase the chance that all students will find a type of learning material that suits their needs. An analysis of the effects of the learning material described here was beyond the scope of the reported study. Further research is needed to investigate how this learning material impacts students’ learning processes. The active student involvement was limited to a group of student representatives. Their opinions might not correspond with other students in the faculty or other nursing faculties, and those differences should also be taken into consideration [58].

## Conclusion

This study indicate that iterative involvement of students in the process of developing new technological learning material enhances student identification of important learning needs. Further, the use of students’ and teachers’ knowledge in an adapted co-design process appears to be the most optimal level of involvement for both students and instructors. Further studies is needed to optimize the approach for student involvement and adjust it to various settings and professions.
